# Supplementary material for: If Cooperation Is Likely Punish Mildly: Insights from Economic Experiments Based on the Snowdrift Game
Source: PLoS One. 2013 May 31;8(5):e64677. doi: 10.1371/journal.pone.0064677 (PMC3669423; doi:10.1371/journal.pone.0064677)
Supplement: Text S1 — Additional information supporting the main conclusions of this paper. The document contains instructions that were given to participants of Treatments I, II and III, details of the regression analysis, and additional results on the evolution of the cooperation level, the rate of strategy changes during the economic experiments, and the percentages of strategies that were employed by the participants. (PDF) [file pone.0064677.s001.pdf]

Supplementary Text S1

The economic experiments were carried out in three treatments with 16 sessions each. There were a total of 320 freshmen and sophomore students involved (45% females, 20.3 years old on average), majoring in mathematics, physics, computer science, economics, as well as different areas of social sciences. Each session was independent and subjects were not allowed to participate in more than one session. In a session, 20 subjects were allocated anonymously to four groups of five subjects. Subjects played the snowdrift game with all their group members. For example, students labeled A, B, C, D, and E were allocated anonymously in the group, then person A played games with person B, C, D, and E simultaneously, obtaining profit according to payoff matrix. Since the previous research shows that punishment works when combination with reputation building is available [1], the groups are unchanged during the whole session. At the same time, each subject does not know who they play games with because subjects were allocated anonymously. At the beginning of the experiment, participants were given printed direction documents to introduce the rule and payoff matrix of the games. After reading the document, subjects were asked to complete some questionnaires to assure them to understand the document correctly. They were then allowed to continue to play games after answer questions correctly. It was emphasized that participants choice the strategy depending on their own decisions. It was forbidden to communicate with the other participants during the experiment. Participants were further informed that every one in the same session had received the same instructions as other participants.

After experiments starting, participants select their strategies on the screen of computers, and their profits in current round were shown on the computer screen. The data of each round was saved automatically by the computer. As shown in Fig. 1, cooperation level was average from two session. In a round of a session, cooperation level is defined as the rate of cooperators among 20 subjects allocated anonymously to four groups. To reduce the effects of beginning and the end, the first two rounds and the last two rounds were abandoned. For example, if there were total 25 rounds, results in this session (see main text) was averaged from 3 to 23 rounds.

Since individuals' decision in the next round is likely according to current round, we define rate to change strategy in given conditions to study how the probability of changing strategy depends on the actions of the others in the group. Here, given conditions involve numbers of cooperative opponent players in the group. We first calculate M, the number of cases of changing strategy with condition of a given number of cooperative opponent players in the group, then count K, the number of cases with the given number of cooperative opponent players in the group. Therefore, the rate to change strategy for the given number of cooperative opponent players in the group is M/K. For example, at $r=0.1$ there were 93 cases where subjects had two cooperative opponent players in their group, and 34 cases that subjects changed their strategies. Therefore, rate to change strategy for two cooperative opponent players is $34/93 \approx 0.366$, as show in Fig. 2(a). It is found that subjects are more likely to change strategy in the next round when they met zero cooperative opponent player for $r=0.6$ and $r=0.9$. In addition, from detailed analysis (not show here) , we found that for condition of $n\_C=2$ at $r=0.1$, 16 cooperative subjects change their strategies to defect in the next round, and 18 defective subjects change their strategies to cooperation (there were total 34 cases of changing strategies). However, there were 6 cooperative subjects to defect in the next round for condition of $n\_C=2$ at $r=0.9$, comparing 1 defective subjects change their strategies to cooperation (there were total 7 cases of changing strategies).

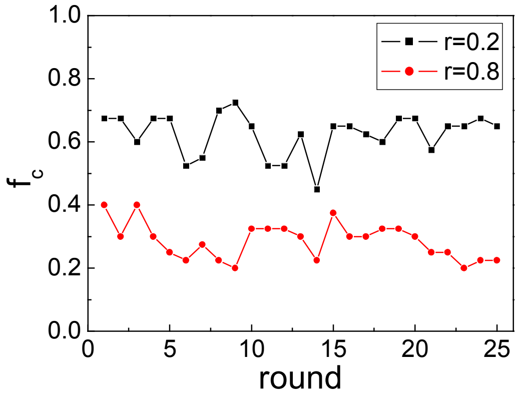

Figure 1: Cooperation level evolving with time in the absence of punishment using cost-to-benefit ratio r=0.2 and r=0.8 in *Treatment I*.

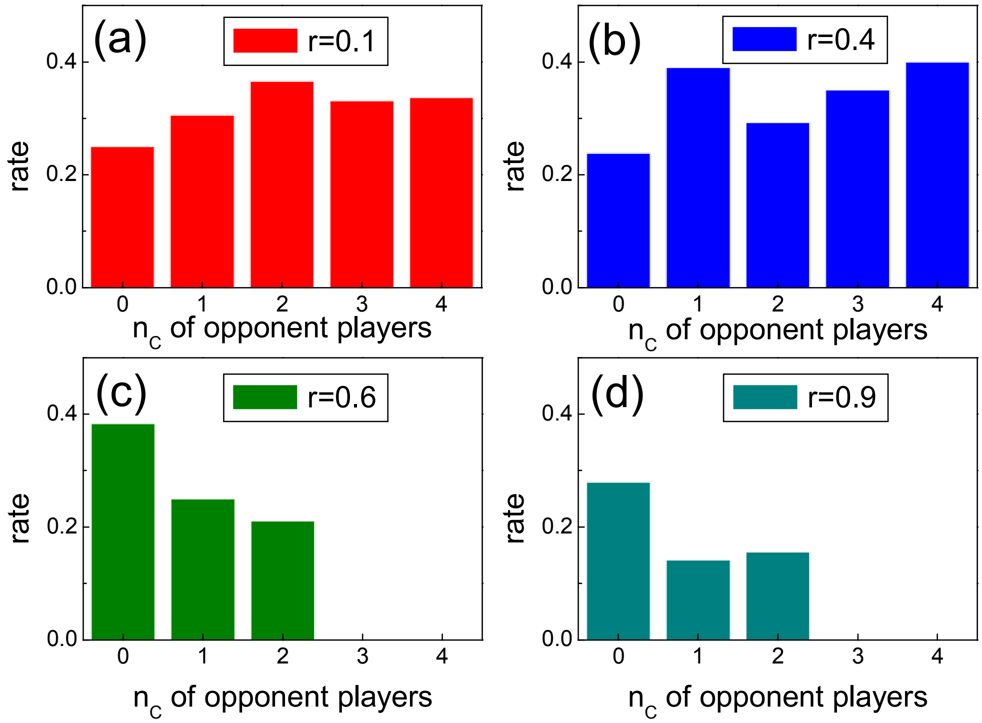

Figure 2: Rate to change strategy in conditions with different number of cooperative opponent players in the group. Individuals' decision in the next round is likely according to current round. We first calculate M, the number of cases of changing strategy with condition of a given number of cooperative opponent players in the group, then count K, the number of cases with condition with the given number of cooperative opponent players in the group. Therefore, the rate to change strategy for the given number of cooperative opponent players in the group is M/K. Data obtained from experiments of *Treatment I*.

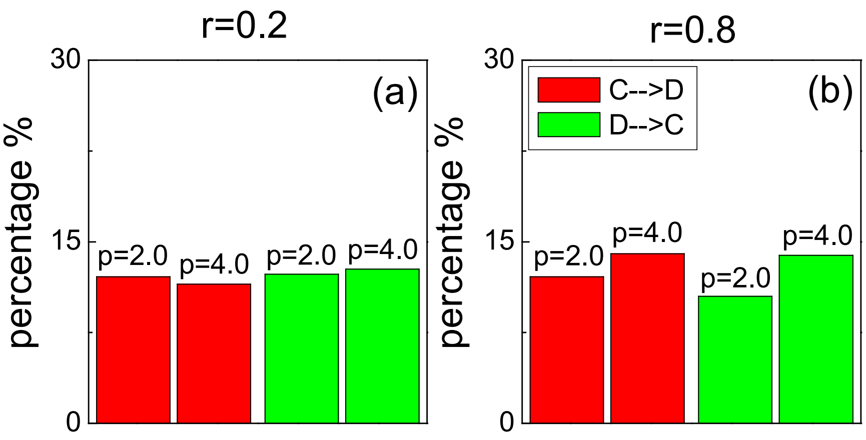

Figure 3: Percentage of cooperators choosing defect and defectors selecting cooperative strategy in conditions of punishment with p=2.0 and p=4.0 at r= 0.2 and r= 0.8, respectively. Data obtained from experiments of *Treatment II*, and *Treatment III* at r=0.2 and r=0.8. The red one presents percentage of cooperators choosing defect in all cases of subjects choosing strategies in the next rounds, and the green one indicates percentage of defectors to cooperate in all cases of subjects choosing strategies in the next rounds.

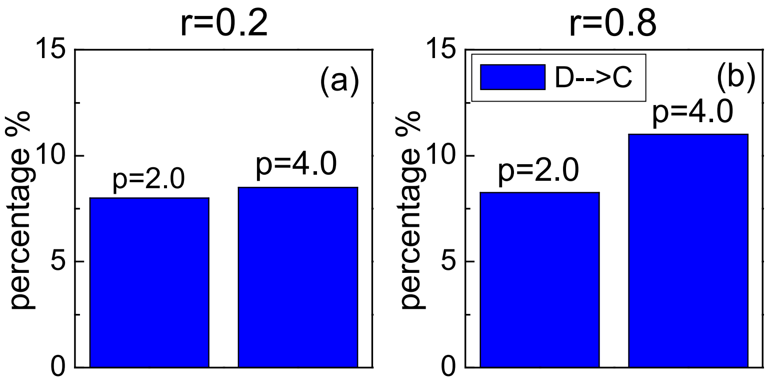

Figure 4: Percentage of defectors selecting cooperative strategy in the next round after being punished in all cases of subjects choosing strategies in conditions of punishment with p=2.0 and p=4.0 at r=0.2 and r=0.8, respectively. Data obtained from experiments of *Treatment II*, and *Treatment III* at r=0.2 and r=0.8.

---

[1] Rockenbach, B. & Milinski, M. The efficient interaction of indirect reciprocity and costly punishment. Nature 444, 718 - 723 (2006).

Table 1：Regression------Effect of the cost-to-benefit ratio r on cooperation level on individual-level data

Source	SS	df	MS
Model	5.131847	1	5.13184745
Residual	9.109434	158	0.05765465
Total	14.24128	159	0.08956781

Number of obs	= 160
F(1, 158)	= 89.01
Prob > F	= 0.0000
R-squared	= 0.3604
Adj R-squared	= 0.3563
Root MSE	= 0.24011

Cooperation level	Coef.	Std. Err.	t	P>|t|	[95% Conf.	Interval]
r	-0.60544	0.0641731	-9.43	0.0000	-0.7321902	-0.47869
_cons	0.738436	0.0372812	19.81	0.0000	0.6648017	0.812069

Table 2：Regression------Effect of the cost-to-benefit ratio r on cooperation level on group-level data

Source	SS	df	MS
Model	1.026368	1	1.02636818
Residual	0.129912	30	0.00433041
Total	1.156281	31	0.03729937

Number of obs	= 32
F(1, 158)	= 237.01
Prob > F	= 0.0000
R-squared	= 0.8876
Adj R-squared	= 0.8839
Root MSE	= 0.06581

Cooperation level	Coef.	Std. Err.	t	P>|t|	[95% Conf.	Interval]
r	-0.60544	0.0393265	-15.4	0.0000	-0.6857575	-0.52513
_cons	0.738435	0.0228467	32.32	0.0000	0.6917762	0.785094

Table 3: Regression------Effect of the cost-to-benefit ratio r on average payoff on individual data

Source	SS	df	MS		
				Number of obs	= 160
				F(1, 158)	= 521.15
Model	109.1741	1	109.174102	Prob > F	= 0.0000
Residual	33.09913	158	0.20948815	R-squared	= 0.7674
Total	142.2732	159	0.89480019	Adj R-squared	= 0.7659
				Root MSE	= 0.4577

average payoff	Coef.	Std. Err.	t	P>|t|	[95% Conf.	Interval]
r	-2.79252	0.1223251	-22.83	0.0000	-3.03412	-2.55091
_cons	4.021258	0.0710645	56.59	0.0000	3.8809	4.161617

Table 4: Regression------Effect of the cost-to-benefit ratio r on average payoff on on group data

Source	SS	df	MS		
				Number of obs	= 32
				F(1, 158)	= 172.66
Model	21.83482	1	21.8348222	Prob > F	= 0.0000
Residual	3.793794	30	0.1264598	R-squared	= 0.8520
Total	25.62862	31	0.82672955	Adj R-squared	= 0.8470
				Root MSE	= 0.35561

average payoff	Coef.	Std. Err.	t	P>|t|	[95% Conf.	Interval]
r	-2.79252	0.2125187	-13.14	0.0000	-3.226538	-2.3585
_cons	4.021258	0.1234622	32.57	0.0000	3.769115	4.273402

Direction documents for Treatment I

The following directions were originally written in Chinese. We document the instructions we have used in the Treatment I for r =0.1. The directions in the other sessions with different values of r were adapted accordingly. They are available upon request.

You are now taking part in an economic game experiment which has been financed by various foundations for research promotion. Please read the following directions carefully, and you can earn a considerable amount of money according your final score. It is worthy mentioning that all 20 participants here have received the same direction document. **You should select the strategy depending on your own decisions. It is forbidden to communicate with the other participants during the experiment.** If you violate this rule, we shall have to exclude you from the experiment and from all payments. There are 20 subjects participating in the economic game experiment, and will be allocated anonymously to four groups of five subjects. Therefore, you will have four group members when the experiment stated, and play games with all your four group members. In the game experiment, each subject has two strategies to choose: cooperation or defect. The names of strategies does not have any morals meaning, and you should choose the strategy the maximize your profit score, because the more score you have the more money you earn in the end of the game. After you choose the strategy in a round, your profit score is sum of all payoff from games played with your four group members according to payoff matrix described in the second paragraph. It is worthy mentioning that after a round you can choose the strategy afresh according to your own decision, and profit score are affected by both your decision and choices of your group members. The total profit score you obtained in the end is the sum of all the profit score of all rounds. One score unit corresponds to 1.0 RMB in our experiment, so you will earn the money which is equal to your final score multiplied by 1.0 RMB when the experiment finished. The payoff matrix of the game carried out here is describe below. Supposing you play the game with a subject labeled A, there are four possible different conditions you may meet, accordingly you obtain different payoffs:

(1) If you cooperate and A defects, then your payoff is 0.9, A’s payoff is 1.1.
(2) If you cooperate and A also cooperates, then both you and A obtain payoff of 1.0.
(3) If you defect and A cooperates, then your payoff is 1.1, A’s payoff is 0.9.
(4) If you defect and A also defects, then both you and A obtain payoff of 0.0.

Since you play games with your four group members, your profit score in this round is sum of all payoff from games played with your four group members according to payoff matrix described above. Supposing you, subject A, subject B, subject C, and subject D in the same group, here some questions you need to answer correct to start experiment:

Q1: If you chose cooperation, subject A and subject B also chose cooperation, subject C and subject D chose defection, then you obtain your profit score in this round__________?

Q2: If you chose defection, subject A and subject B also chose cooperation, subject C and subject D chose defection, and then you obtain your profit score in this round__________?

Q3: If your total profit score at the end of the game is 50.0, how much we should pay cash to you__________RMB?

Direction documents for Treatment II and Treatment III

The following directions were originally written in Chinese. We document the instructions we have used in the Treatment II for r = 0.2 with punishment p = 2.0. The directions in the other sessions and treatments were adapted accordingly. They are available upon request.

You are now taking part in an economic game experiment which has been financed by various foundations for research promotion. Please read the following directions carefully, and you can earn a considerable amount of money according your final score. It is worthy mentioning that all 20 participants here have received the same direction document. **You should select the strategy depending on your own decisions. It is forbidden to communicate with the other participants during the experiment.** If you violate this rule, we shall have to exclude you from the experiment and from all payments. There are 20 subjects participating in the economic game experiment, and will be allocated anonymously to four groups of five subjects. Therefore, you will have four group members when the experiment stated, and play games with all your four group members. It is worthy mentioning that after a round you can choose the strategy afresh according to your own decision, and profit score are affected by both your decision and choices of your group members. The total profit score you obtained in the end is the sum of all the profit score of all rounds. One score unit corresponds to 1.0 RMB in our experiment, so you will earn the money which is equal to your final score multiplied by 1.0 RMB when the experiment finished. There are two stages in this experiment, and the profit score in a round is sum of payoffs in first stage and the second stage.

The first stage

In the first stage, each subject has two strategies to choose: cooperation or defect. The names of strategies does not have any morals meaning, and you should choose the strategy the maximize your profit score, because the more score you have the more money you earn in the end of the game. After you choose the strategy in a round, your profit score is sum of all payoff from games played with your four group members according to payoff matrix described in the second paragraph. The payoff matrix of the game carried out here is described below. Supposing you play the game with a subject labeled A, there are four possible different conditions you may meet, accordingly you obtain different payoffs:

(1) If you cooperate and A defects, then your payoff is 0.8, A’s payoff is 1.2.

(2) If you cooperate and A also cooperates, then both you and A obtain payoff of 1.0.

(3) If you defect and A cooperates, then your payoff is 1.2, A’s payoff is 0.8.

(4) If you defect and A also defects, then both you and A obtain payoff of 0.0.

Since you play games with your four group members, your profit score in this round is sum of all payoff from games played with your four group members according to payoff matrix described above in the first stage and payoff of obtained below in the second stage.

The second stage

In second stage, cooperators can choose punishment strategy or non-punishment strategy if there are defectors in the group where she/he belongs.

Conditions I: If there is at least one cooperator among 5 group members who choose punishment strategy in this stage, the payoffs of all defectors in this group are reduced by 2 (fine for being punished), and cooperators who take punishment strategy loose 1 unit (cost of punishment), while payoff of cooperators who take non-punishment strategy remain unchanged.

Conditions II: If there are no cooperators who take punishment strategy, payoffs of cooperators and defectors remain unchanged.
Conditions III: If all group members in the group are cooperators or defectors, there is no punishment and payoffs remain unchanged.

Supposing you, subject A, subject B, subject C, and subject D in the same group, here some questions you need to answer correct to start experiment:

Q1: In the first stage, if you chose cooperation, subject A and subject B also chose cooperation,subject C and subject D chose defection. In the second stage if you chose punishment strategy, subject A also chose punishment strategy, while subject B chose non-punishment strategy. You obtain your profit score in this round__________? Subject A obtains profit score in this round__________? Subject B obtains profit score in this round __________? Subject C obtains profit score in this round __________? Subject D obtains profit score in this round __________?

Q2: In the first stage, if you chose cooperation, subject A and subject B also chose cooperation, subject C and subject D chose defection. In the second stage if you chose non-punishment strategy, subject A chose punishment strategy, subject B chose non-punishment strategy. You obtain your profit score in this round __________? Subject A obtains profit score in this round __________? Subject B obtains profit score in this round. __________? Subject C obtains profit score in this round__________? Subject D obtains profit score in this round __________?

Q3: In the first stage, if you chose cooperation, subject A and subject B also chose cooperation, subject C and subject D chose defection. In the second stage if you chose non-punishment strategy, subject A chose nonpunishment strategy, subject B chose non-punishment strategy. You obtain your profit score in this round __________? Subject A obtains profit score in this round __________? Subject B obtains profit score in this round __________? Subject C obtains profit score in this round . . . . ? Subject D obtains profit score in this round __________?

Q4: In the first stage, if you chose cooperation, subject A, subject B, subject C, and subject D also chose cooperation. You obtain your profit score in this round . . . . ? Subject A obtains profit score in this round __________? Subject B obtains profit score in this round __________? Subject C obtains profit score in this round__________? Subject D obtains profit score in this round __________?

Q5: In the first stage, if you chose defection, subject A subject B subject C and subject D also chose defection. You obtain your profit score in this round __________? Subject A obtains profit score in this round__________? Subject B obtains profit score in this round __________? Subject C obtains profit score in this round __________? Subject D obtains profit score in this round__________?

Q6: If your total profit score at the end of the game is 50.0, how much we should pay cash to you __________RMB?
